# Anxiety, Depression, Schizophrenia, and Bipolar Disorder in Eosinophilic Esophagitis Patients: A Global Federated Cohort Analysis of Bidirectional Risks

**DOI:** 10.1111/all.70288

**Published:** 2026-03-12

**Authors:** Hui‐Chin Chang, Meng‐Che Wu, Torsten Zuberbier, Shuo‐Yan Gau

**Affiliations:** ^1^ Evidence‐Based Medicine Center Chung Shan Medical University Hospital Taichung Taiwan; ^2^ Library Chung Shan Medical University Hospital Taichung Taiwan; ^3^ School of Medicine Chung Shan Medical University Taichung Taiwan; ^4^ Division of Pediatric Gastroenterology, Children's Medical Center Taichung Veterans General Hospital Taichung Taiwan; ^5^ Department of Post‐Baccalaureate Medicine, College of Medicine National Chung Hsing University Taichung Taiwan; ^6^ Institute of Allergology, Charité‐Universitätsmedizin Berlin, Corporate Member of Freie Universität Berlin and Humboldt‐Universität Zu Berlin Berlin Germany; ^7^ Fraunhofer Institute for Translational Medicine and Pharmacology, Immunology and Allergology Berlin Germany; ^8^ Department and Graduate Institute of Business Administration National Taiwan University Taipei Taiwan; ^9^ Orthopedics Department Chi‐Mei Medical Center Tainan Taiwan; ^10^ Department of Medical Education Ditmanson Medical Foundation Chia‐Yi Christian Hospital Chiayi City Taiwan

**Keywords:** clinical immunology, eosinophils, epidemiology, esophagitis, inflammation

To the Editor,

Eosinophilic esophagitis (EoE) impairs quality of life through persistent symptoms and long‐term dietary management [1]. Emerging studies suggest EoE is associated with increased psychiatric morbidity, but evidence is mostly limited to anxiety and depression in single‐country cohorts, with little data on broader outcomes globally [2, 3, 4]. We therefore conducted a large, multinational cohort study to evaluate psychiatric risks associated with EoE.

This retrospective cohort study was derived from the TriNetX Global Collaborative Network, an electronic health record platform utilized in many epidemiological studies [5]. Adults aged ≥ 18 years with at least two visits between January 2005 and December 2023 were eligible. Patients with EoE were identified using ICD‐10‐CM codes (Table S1), and individuals without a diagnosis of EoE served as controls; those with prior death, malignancy, or pre‐existing psychiatric disorders were excluded. Propensity score matching (1:1) was applied to balance age, sex, race, body mass index, atopic comorbidities, cardiometabolic diseases, substance use disorders, healthcare utilization, and socioeconomic indicators. The primary outcomes were incident generalized anxiety disorder, major depressive disorder (MDD), bipolar disorder, and schizophrenia occurring ≥ 3 months after the index date. Hazard ratios (HRs) with 95% confidence intervals (CIs) were estimated using Cox proportional hazards models. Sensitivity analytic models were reported in Table S2. Bidirectional analyses assessing the risk of EoE following psychiatric disorders were also performed.

After matching, 39,331 well‐balanced patients were included in each cohort (Table 1; Figure S1). During follow‐up, EoE was associated with a higher risk of generalized anxiety disorder (HR 1.34, 95% CI 1.20–1.50) and MDD (HR 1.67, 95% CI 1.44–1.93) compared with controls (Figure S2). These associations were generally consistent across sex and age subgroups and robust in sensitivity analyses. When compared with patients with gastroesophageal reflux disease, the risk estimates for anxiety and MDD were attenuated (Figure 1). No consistent association was found between EoE and schizophrenia, with low event rates and wide confidence intervals. For bipolar disorder, an elevated risk in crude analyses was attenuated after comprehensive matching and varied across analytic strategies. In bidirectional analyses, prior generalized anxiety disorder (HR 1.23, 95% CI 1.05–1.44) and MDD (HR 1.57, 95% CI 1.31–1.89) were associated with an increased subsequent risk of EoE, whereas bipolar disorder was not (Figure S3).

**TABLE 1:** Baseline characteristics of study subjects (before and after propensity score matching).

	Before matching			After matching^a^		
	EoE cohort (*n* = 39,335)	Control cohort (*n* = 6,934,892)	SMD	EoE cohort (*n* = 39,331)	Control cohort (*n* = 39,331)	SMD
Age at index						
Mean ± SD	32.6 ± 17.3	39.0 ± 20.4	0.34	32.6 ± 17.3	32.6 ± 17.3	0.00
Sex						
Male	24,688 (62.8)	3,053,361 (44.3)	0.38	24,686 (62.8)	25,106 (63.8)	0.02
Female	13,256 (33.7)	3,610,827 (52.4)	0.39	13,256 (33.7)	13,233 (33.6)	0.00
Unknown Gender	5174 (13.2)	1,717,838 (25.0)	0.30	5174 (13.2)	4255 (10.8)	0.07
Race, *n* (%)						
White	30,081 (76.5)	3,445,919 (50.1)	0.57	30,079 (76.5)	30,076 (76.5)	0.00
Black or African American	1864 (4.7)	870,106 (12.6)	0.28	1864 (4.7)	2759 (7.0)	0.10
Asian	617 (1.6)	393,217 (5.7)	0.22	617 (1.6)	1000 (2.5)	0.07
American Indian or Alaska Native	110 (0.3)	21,907 (0.3)	0.01	110 (0.3)	67 (0.2)	0.02
Native Hawaiian or other Pacific Islander	95 (0.2)	32,810 (0.5)	0.04	95 (0.2)	91 (0.2)	0.00
Other Race	1392 (3.5)	403,002 (5.9)	0.11	1392 (3.5)	1083 (2.8)	0.05
Unknown Race	1389 (3.5)	220,611 (3.2)	0.02	1389 (3.5)	992 (2.5)	0.06
Socioeconomic status						
Persons with potential health hazards related to socioeconomic and psychosocial circumstances	132 (0.3)	28,589 (0.4)	0.01	132 (0.3)	112 (0.3)	0.01
Lifestyle						
Mental and behavioral disorders due to psychoactive substance use	10 (< 0.1)	98 (< 0.1)	0.02	10 (< 0.1)	0 (< 0.1)	0.02
Comorbidities						
Hypertension	2658 (6.8)	677,276 (9.8)	0.11	2658 (6.8)	2627 (6.7)	0.00
Hyperlipidemia	1896 (4.8)	368,261 (5.3)	0.02	1896 (4.8)	1891 (4.8)	0.00
Diabetes mellitus	873 (2.2)	280,057 (4.1)	0.11	873 (2.2)	866 (2.2)	0.00
Chronic kidney disease	260 (0.7)	77,681 (1.1)	0.05	260 (0.7)	235 (0.6)	0.01
Chronic ischemic heart disease	414 (1.1)	121,802 (1.8)	0.06	414 (1.1)	424 (1.1)	0.00
Asthma	4224 (10.7)	220,991 (3.2)	0.30	4222 (10.7)	4194 (10.7)	0.00
Alergic rhinitis	3716 (9.4)	269,115 (3.9)	0.22	3714 (9.4)	3702 (9.4)	0.00
Celiac disease	384 (1.0)	5956 (0.1)	0.12	384 (1.0)	54 (0.1)	0.11
Atopic dermatitis	512 (1.3)	33,608 (0.5)	0.09	511 (1.3)	480 (1.2)	0.01
Medical utilization status						
Visit: Ambulatory	27,206 (69.2)	3,870,950 (56.2)	0.27	27,205 (69.2)	27,232 (69.2)	0.00
Visit: Inpatient Encounter	4289 (10.9)	773,795 (11.2)	0.01	4289 (10.9)	4297 (10.9)	0.00
BMI, *n* (%)						
≧ 25 (kg/m^2^)	10,682 (27.2)	1,216,981 (17.7)	0.23	10,681 (27.2)	10,736 (27.3)	0.00

Abbreviations: EoE, eosinophilic esophagitis; SMD, standardized mean difference.

^a^ In the TriNetX analytics platform, for deidentification purposes, if the number of incident cases is 10 or fewer, it will be reported as 10. Propensity score matching was performed on age at index, sex, race, body mass index, status of comorbidities (including diabetes mellitus, hypertension, hyperlipidemia, atopic dermatitis, allergic rhinitis, asthma), status of smoking, alcoholism and substance use (mental and behavioral disorders due to psychoactive substance use), medical utilization status (ambulatory and acute inpatient visit) and socioeconomic status (problems related to housing and economic circumstances, persons with potential health hazards related to socioeconomic and psychosocial circumstances).

**FIGURE 1:**
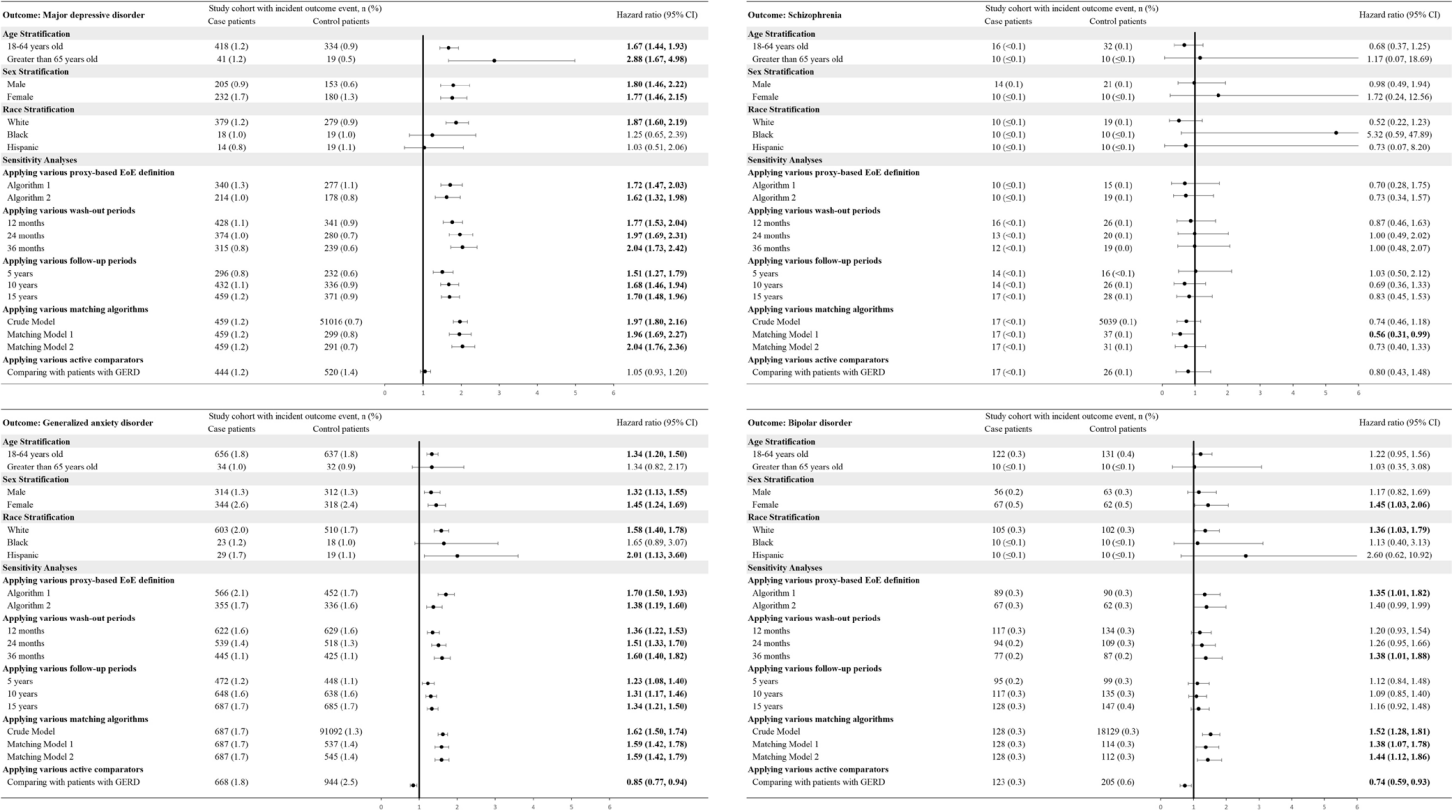
Risk of major depressive disorder, generalized anxiety disorder, schizophrenia, and bipolar disorder in eosinophilic esophagitis patients under different models and stratification statuses.

We report that EoE was associated with an increased risk of common mood and anxiety disorders. Associations with schizophrenia were not robust, and estimates for bipolar disorder varied across analytic strategies, likely reflecting low event counts and limited statistical power. These findings extend prior single‐country studies and suggest that psychiatric burden in EoE predominantly involves anxiety‐depressive domains [3]. From a real‐world perspective, severe mental illnesses such as schizophrenia are less clearly related to atopic or inflammatory conditions [6]; however, these negative findings should be interpreted with caution, as they may largely reflect low event counts rather than a true absence of association. Potential mechanisms may involve persistent disease‐related burden and lifestyle constraints, which may preferentially predispose patients to anxiety and depressive disorders rather than severe psychiatric conditions.

Though the bidirectional design and multinational scope provide a more integrated real‐world perspective on the psychiatric burden associated with EoE, several limitations should be acknowledged. As an observational analysis based on administrative codes, causal inference cannot be established, and residual confounding related to disease severity, symptom burden, dietary restriction, and psychosocial stress may persist. Subclinical or undiagnosed psychiatric conditions before cohort entry may also be incompletely captured. Moreover, although data were drawn from multiple countries, findings may not be fully generalizable beyond healthcare‐engaged populations. These findings highlight the importance of heightened awareness and timely screening for anxiety and depressive disorders in patients with EoE as part of comprehensive disease management.

## Author Contributions

All the authors involved in drafting or revising the article approved of the submitted version. Study conception and design: Shuo‐Yan Gau, Hui‐Chin Chang, Meng‐Che Wu, and Torsten Zuberbier. Data acquisition: Hui‐Chin Chang and Shuo‐Yan Gau. Data analysis and demonstration: Shuo‐Yan Gau, Hui‐Chin Chang, Meng‐Che Wu, and Torsten Zuberbier. Original draft preparation: Shuo‐Yan Gau, Hui‐Chin Chang, Meng‐Che Wu, and Torsten Zuberbier.

## Funding

This study was partially supported by the funding from Chung Shan Medical University Hospital (CSH‐2025‐C‐007) and the Taiwan Global Pathfinders Initiative (Program No: 190).

## Ethics Statement

This research has been approved by the Institutional Review Board of Chung Shan Medical University Hospital (IRB No. CS1‐25002) and the need of informed consent is exempted by the Institutional Review Board. Trial registry is not applicable as the current study is not a clinical trial.

## Consent

The authors have nothing to report.

## Conflicts of Interest

The authors declare no conflicts of interest.

## Supporting information


**Table S1:** Utilized proxy codes ^a^.
**Table S2:** Description of all applied sensitivity analysis models in Figures 2–6.
**Figure S1:** Patient selection flowchart.
**Figure S2:** Cumulative probability of generalized anxiety disorder, major depressive disorder, schizophrenia and bipolar disorder risk in EoE patients.
**Figure S3:** Risk of eosinophilic esophagitis in generalized anxiety disorder, major depressive disorder, bipolar disorder and schizophrenia.

## Data Availability

Data in this study were retrieved from TriNetX Research Network. All data available in the database were administered by the TriNetX platform. Detailed information can be retrieved at the official website of the research network (https://trinetx.com).
