# Supplementary material for: Anxiety, Depression, Schizophrenia, and Bipolar Disorder in Eosinophilic Esophagitis Patients: A Global Federated Cohort Analysis of Bidirectional Risks
Source: Allergy. 2026 Mar 12;81(9):3361–4. doi: 10.1111/all.70288 (PMC13569653; doi:10.1111/all.70288)
Supplement: Supplementary file 1 [file ALL-81-3361-s001.docx]

**Supplementary Files**

**Table S1.** Utilized proxy codes ^a^

| **Description** | **ICD-10-CM codes** |
| --- | --- |
| *Study population* |  |
| Eosinophilic esophagitis | ICD-10-CM: K20.0 |
| *Outcome events* |  |
| Generalized anxiety disorder | ICD-10-CM: F41.1 |
| Major depressive disorder | ICD-10-CM: F33 |
| Schizophrenia | ICD-10-CM: F20 |
| Bipolar disorder | ICD-10-CM: F31 |
| Suicide | ICD-10-CM: F48, T14.91 |
| *Covariates and other definitions* |  |
| Essential hypertension | ICD-10-CM: I10 |
| Hyperlipidemia | ICD-10-CM: E78.5 |
| Diabetes mellitus | ICD-10-CM: E08-E15 |
| Chronic ischemic heart disease | ICD-10-CM: I25 |
| Chronic kidney disease | ICD-10-CM: N18 |
| Asthma | ICD-10-CM: J45 |
| Vasomotor and allergic rhinitis | ICD-10-CM: J30 |
| Celiac disease | ICD-10-CM: K90.0 |
| Atopic dermatitis | ICD-10-CM: L20 |
| Persons with potential health hazards related to socioeconomic and psychosocial circumstances | ICD-10-CM: Z55-Z65 |
| Mental and behavioral disorders due to psychoactive substance use | ICD-10-CM: F10-F19 |
| Encounter for general examination | ICD-10-CM: Z00 |

^a^ICD-10-CM: International Classification of Diseases, Tenth Revision, Clinical Modification

**Table S2.** Description of all applied sensitivity analysis models in Figures 2-6

| **Models** | **Description** |
| --- | --- |
| **Applying various proxy-based eosinophilic esophagitis (EoE) definition** | |
| Algorithm 1 | Only patients with the record of ICD-10-CM codes of K20.0 (eosinophilic esophagitis) with prescription record of proton pump inhibitors (ATC code: A02BC) were included as EoE group in this model. |
| Algorithm 2 | Only patients with the record of ICD-10-CM codes of K20.0 (eosinophilic esophagitis) with record of esophagogastroduodenoscopy procedures (CPT code: 1021431) were included as EoE group in this model. |
| **Applying different wash-out period after index date** | |
| 12 months/24 months/36 months after index date | Any outcome events occurring during the designated washout period were excluded from subsequent analyses. For all subgroup analyses, the follow-up duration was standardized to 15 years. |
| 5 years /10 years /15 years after index date | Only outcome events occurring within the defined follow-up period were included in the analysis. In all subgroup analyses, a washout period of 3 months was applied. |
| **Applying different matching covariates** | |
| Crude model | The hazard ratio was estimated using the population prior to the application of propensity score matching. |
| Matching model 1 | Matching covariates include age at index and sex |
| Matching model 2 | Matching covariates include age at index, sex, socioeconomic status and substance abuse status |
| **Applying different active comparators** | |
| Comparing with Gastroesophageal Reflux Disease patients | The control cohort in this sub-analysis consisted of patients with gastroesophageal reflux disease, which has been reported to be associated with psychiatric disorders. In all sub-analyses, the follow-up period was set as 15 years and the wash-out period was set as 3 months. |

**Supplementary figures**

**Figure S1.** Patient selection flowchart

MDD

SCZ

BD

GAD

**Figure S2.** Cumulative probability of generalized anxiety disorder, major depressive disorder, schizophrenia and bipolar disorder risk in EoE patients

*Legends:*

*EoE, eosinophilic esophagitis; GAD, generalized anxiety disorder; MDD, major depressive disorder, BD, bipolar disorder, SCZ, schizophrenia*

**Figure S3.** Risk of eosinophilic esophagitis in generalized anxiety disorder, major depressive disorder, bipolar disorder and schizophrenia
